# Effects of short-term extreme temperature treatment on the development and reproductive capacity of *Encarsia formosa*

**DOI:** 10.3389/fphys.2023.1187743

**Published:** 2023-05-30

**Authors:** Ming-Jiang Li, Bo Zhang, Guo-Hua Chen, Shun-Wen Zhou, Ji-Huan Liu, Mei Lu, Jin-Long Zhang, Shao-Wu Yang, Xiao-Ming Zhang

**Affiliations:** ^1^ National Key Laboratory for Conservation and Utilization of Biological Resources in Yunnan, College of Plant Protection, Yunnan Agricultural University, Kunming, China; ^2^ Yunnan Yuntianhua Co., Ltd., Kunming, Yunnan, China; ^3^ College of Resources and Environment, Yunnan Agricultural University, Kunming, China; ^4^ Yunnan Urban Agricultural Engineering and Technological Research Center, College of Agronomy and Life Sciences, Kunming University, Kunming, China

**Keywords:** Bemisia tabaci, Encarsia formosa, extreme temperature, development, reproductive capacity

## Abstract

*Encarsia formosa* is a natural enemy of the invasive pest *Bemisia tabaci* and is known to be a dominant parasitic. The frequency and magnitude of climate extremes, particularly temperature extremes, have increased, which has put insect populations at risk. However, the effects of temperature extremes on *E. formosa* are not well understood. To examine the impact of short-term extreme temperature exposure on the development and reproduction of *E. formosa*, eggs, larvae, pupae, and adults were exposed to high/low temperature treatments (HLT_25_, HLT_50_, LLT_25_, and LLT_50_). Our findings indicate that the pupal stage of *E. formosa* exhibited the strongest tolerance to both heat and cold, while adults exhibited a weaker tolerance. The shortest egg-to-adult development period of 12.65 days was observed in *E. formosa* exposed to HLT_50_ treatment during the egg-larval stage. The parasitism peak of the adult stage was delayed by 1–6 days after exposure to extreme temperatures during the egg-larval stage. Conversely, the parasitism peak was advanced by 1–3 days after exposure to extreme temperatures during the pupal and adult stages. The eclosion rate, total parasitism, eclosion rate of the F_1_ generation, and adult longevity of the F_1_ generation were lower in the treatment groups than in the control groups. The F_1_ generation’s development period was prolonged to 15.49 and 15.19 days after exposure to HLT_25_ and HLT_50_ treatments, respectively, during the egg-larval stage. The F_1_ generation’s development period was shortened to 13.33 days after exposure to LLT_50_ treatment during the pupal stage. Male individuals appeared in the F_1_ generation after exposure to HLT_50_ treatment during the pupal stage, with females accounting for only 56.38%. Our results demonstrate that short-term exposure to extreme temperatures has detrimental effects on the growth and reproduction of *E. formosa*. In field biocontrol against *E. formosa*, the release of *E. formosa* should be avoided as much as possible when the ambient temperature is higher than 35°C or lower than 0°C. During extreme temperature conditions, timely supplementation and release of *E. formosa* population, along with ventilation and cooling in greenhouse facilities during summer, are necessary for better pest control efficacy.

## Highlights

1) *E. formosa* exhibits varied tolerance to temperature extremes depending on its developmental stage. The pupal stage of *E. formosa* demonstrated a high degree of tolerance to both heat and cold, whereas the adult stage displayed weaker tolerance. 2) Exposure to short-term temperature extremes had detrimental effects on the eclosion rate, total parasitism, eclosion rate of the F_1_ generation, and adult longevity of the F_1_ generation in *E. formosa.* 3) Notably, the emergence of male individuals in the F_1_ generation was observed only after exposing the pupal stage of *E. formosa* to HLT_50_ treatment.

## 1 Introduction

*Bemisia tabaci* (Gennadius) (Hemiptera: Aleyrodidae) is a highly polyphagous insect pest characterized by small individuals, diverse biotypes, and a global distribution excluding Antarctica (Kanakala and Ghanim, 2019; Pan et al., 2021; Isaac et al., 2023). This pest’s host plant range is vast, as it can damage over 900 host plant species, including *Gossypium* spp. (Malvales: Malvaceae), *Solanum lycopersicum* Miller (Tubiflorae: Solanaceae), and *Phaseolus vulgaris* L. (Rosales: Fabaceae) (Lee, 2020). *Bemisia tabaci* inflicts direct harm on crops by extracting plant sap and excreting honeydew, which leads to sooty mold and significantly impairs plant photosynthesis. Indirect damage also occurs through the transmission of various plant viruses (Li et al., 2022). The economic losses from indirect damage considerably exceed those caused by direct feeding (Zhang X M et al., 2020). At present, chemical control is the primary method for managing *B. tabaci*. However, the extended use of chemical pesticides heightens the likelihood of resistance development in *B. tabaci* populations (Basit, 2019). Consequently, the implementation of biological control as part of an integrated management strategy for *B. tabaci* is essential (Lenteren et al., 2018; Roda et al., 2020). *Encarsia formosa* Gahan (Hymenoptera: Aphelinidae) is a crucial parasitic of whitefly pests and one of the predominant parasitic species of *B. tabaci*, which may effectively control the population growth of *B. tabaci* (He et al., 2019; Deeksha et al., 2023). *E. formosa* undergoes complete metamorphosis, with the larval and pupal stages developing inside the body of *B. tabaci* (Wang et al., 2019). The adult *E. formosa* have reddish-brown ocelli and rod-shaped antennae, with males having yellow heads and black abdomens, distinguishing them from females (Li, 2017). *E. formosa* is a parthenogenetic parasitic wasp that tends to parasitize the 3rd-instar and 4th-instar nymphs of whitefly, with parasitized 3rd-instar nymphs of *B. tabaci* turning black after about a week (Dai et al., 2014; Yang et al., 2023). Commercial production of *E. formosa* has been established, and it has demonstrated considerable effectiveness in controlling whitefly pests in Hebei, Henan, Liaoning, Shanxi, and Beijing, China (Liu, 2017).

As ectothermic animals, insects are particularly susceptible to external factors such as temperature (Daniel et al., 2020). Temperature stress outside the optimal range may cause changes in insect metabolism, material composition, and hormone levels, negatively impacting their development, reproduction, and survival (Bennett and Moran, 2015). Temperature effects on insects can be classified into two types: long-term and short-term high or low temperature stress, which have distinct effects (Wang et al., 2010). The increasing occurrence of extreme temperature conditions due to global warming poses a significant threat to the survival and reproduction of insects, even for brief periods (Meehl and Tebaldi, 2004). Different developmental stages of insects have different levels of temperature tolerance. For example, in *Propylaea japonica* (Thunberg) (Coleoptera: Coccinellidae), the pupal stage exhibits the highest tolerance to high temperatures, followed by older and younger larvae stages (Pumhan et al., 2020). Cold resistance is the strongest in *B. tabaci* pupae, followed by eggs, nymphs, and adults, with increased mortality as low-temperature stress increases (Yang et al., 2021). Temperature also affects the parasitism of insects, a critical reference index for the control potential of natural pest enemies. High-temperature stress significantly reduces the fecundity of *Trichogramma dendrolimi* Matsumura (Hymenoptera: Trichogrammatidae) females and inhibits their parasitism (Liu et al., 2021), whereas low-temperature storage of adult *Diadegma semiclausum* (Hellén) (Hymenoptera: Ichneumonidae) reduces parasitism and emergence rates (Zhang et al., 2022). Short-term extremely high temperatures may lead to reduced individual fitness of parasitic wasps, causing delayed development, shorter lifespan, reduced fecundity, and decreased female ratio (Klockmann et al., 2017a; Klockmann et al., 2017b). The growth, development, and fecundity of *Aphidius gifuensis* Ashmead (Hymenoptera: Aphidiidae) are seriously impacted by decreasing temperatures (Izumi and Makoto, 2006). *E. formosa* grows optimally within the 15°C–30°C temperature range, and deviation from this range may negatively affect their development and reproduction, ultimately impacting their population growth (Chown, 2006; Yin et al., 2018). However, the effects of extreme temperature on different developmental stages of *E. formosa* remain poorly understood.

Employing *E. formosa* to manage *B. tabaci* populations has become a focal point of contemporary research. Factors such as high summer temperatures, elevated greenhouse temperatures, extremely low winter temperatures, and low-temperature storage technology during commercial production may potentially affect the survival and reproduction of *E. formosa*, thereby altering its efficacy in controlling *B. tabaci*. In this study, we investigated the impacts of short-term exposure to various extreme temperatures on the growth, development, reproductive capacity, and pest control ability of *E. formosa* across its different developmental stages. The insights gained from this research are anticipated to inform the strategic release of *E. formosa* in future agricultural practices and provide a valuable reference for preserving and predicting *E. formosa*’s control efficiency in extreme temperature environments.

## 2 Materials and methods

### 2.1 Insects, host plant, and temperature measurements

The study utilized *E. formosa* insects collected from *P. vulgaris* plants (102°40′E, 25°11′N) in the northern suburb of Kunming City, Yunnan Province, China. The MED cryptic species of *B. tabaci* was kindly provided by the Biotechnology and Germplasm Resources Institute, Yunnan Academy of Agricultural Sciences, and reared separately in insect cages (60 cm long × 60 cm wide × 60 cm deep) with *P. vulgaris* as the host plant for at least 20 generations in an insect-rearing room maintained at a temperature of 25°C ± 1°C and a humidity level of 75% ± 5%. To ensure the purity of the insect populations and minimize external factors, the tested *B. tabaci* and *E. formosa* populations were reared separately in different insect cages in a greenhouse without the use of insecticides for over 20 generations. When we want to use it, we take the appropriate age of insects to test. For the experiment, a single *P. vulgaris* plant was grown in a flowerpot (10 cm in diameter, 12 cm in height) inside an insect cage maintained at a temperature of 25°C ± 1°C and a humidity level of 75% ± 5% in the laboratory. The amount of *P. vulgaris* planted should ensure that the experiment is carried out properly. After the expansion of the first pair of primary leaves in the seedlings, fours pots of seedlings were used per cage, with 200 individuals of *B. tabaci* (male to female ratio of about 1:1) inoculated for oviposition on each cage. After 24 h, the bean seedlings were removed and placed inside an insect cage without the adults of *B. tabaci* for use in experiments after the *B. tabaci* nymphs on the back of the leaves reached the 3rd-instar stage.

For the short-term high-temperature stress treatment, the *E. formosa* insects were subjected to temperatures of 30°C, 34°C, 38°C, 42°C, 46°C, and 50°C. The short-term low-temperature stress treatments were conducted at temperatures of −12°C, −8°C, −4°C, 0°C, 4°C, and 8°C, and temperatures below 0 °C were achieved using a BCD-201STPA refrigerator, while the remaining temperatures were maintained inside a BIC-300 artificial climate chamber. The control-group temperature was 25°C. Each temperature treatment had three replicates, and each stress treatment lasted for 6 h. After exposure to stress, the *E. formosa* insects were immediately transferred to an artificial climate chamber maintained at a temperature of 25°C ± 1°C, a photoperiod of 14:10 (L:D) h, and a humidity level of 75% ± 5% for feeding.

The experimental equipment consisted of a BIC-300 artificial climate chamber (Shanghai Boxun Medical Equipment Factory), a BCD-201STPA refrigerator (Haier Group), and an OLYMPUS SZ51 stereomicroscope (Olympus, Japan).

The insect-rearing containers utilized in the experiments were custom-made. Cylindrical transparent plastic pots, measuring 9 cm in diameter and 5 cm in height, were procured from Hangzhou Wangyi E-commerce Co. Ltd. The detachable lid of the plastic container connects to the main body through a spiral jaw mechanism. The center of the lid was removed and replaced with a 120-mesh screen, which was affixed using hot melt adhesive to enable proper ventilation. These custom-made insect-rearing containers were employed for housing *E. formosa* at various developmental stages during treatment, and for the daily replacement of host plant leaves hosting 50 individual 3^rd^-instar stage nymphs of *B. tabaci*.

### 2.2 Determination of the mortality of *Encarsia formosa* at different developmental stages after short-term temperature treatment

Treatment of the different developmental stages of *E. formosa*:

Adult: *E. formosa* adults within 24 h of emergence;

Pupa: 3 days after the pupation of *E. formosa*;

Egg–larvae: The 3rd day after the 3rd-instar stage nymphs of *B. tabaci* were parasitized by *E. formosa*.

Thirty *E. formosa* specimens at various developmental phases (egg-larvae, pupa, adult) were placed in artificial climate chambers or refrigerators, depending on the required treatment, at distinct high/low-temperature stress treatments or 25°C (control), and subsequently transferred to artificial climate chambers. Each treatment had a duration of 6 h. To preserve leaf freshness, water was added to the petiole. Leaves were examined every 24 h using a stereoscope (OuYang et al., 2014). The number of deceased *E. formosa* adults after 6 h exposure to varying temperatures was documented. For the treatment involving *E. formosa* adults, they were gently touched with a fine brush, and unresponsive individuals were considered dead. The number of emerged and dead *E. formosa* pupae after exposure to different temperatures was recorded. Dead *E. formosa* pupae were identified by their loss of water and shriveled appearance. The number of *E. formosa* pupae, emerged *B. tabaci* adults, and dead *B. tabaci* nymphs after egg-larvae exposure to various temperatures was also noted. For the treatment on *E. formosa* egg-larvae, *B. tabaci* nymphs displaying water loss and withering, as well as the *E. formosa* egg-larvae, were deemed dead (Jiang et al., 2018). Each temperature treatment experiment was conducted three times. The corrected mortality rate of *E. formosa* at different developmental stages was calculated after 6 h of exposure to various temperatures. The findings revealed that extreme-high temperatures (HLT_25_ and HLT_50_) and extreme-low temperatures (LLT25 and LLT_50_) resulted in mortality rates of 25% and 50%, respectively (Zhou et al., 2011).

### 2.3 Effects of short-term exposure to extreme temperature on the growth and development of *Encarsia formosa*

Short-term extreme temperature treatment of adults:

In this study, 30 adult individuals of *E. formosa* were chosen within 24 h of emergence and exposed to extreme temperatures of HLT_25_, HLT_50_, LLT_25_, and LLT_50_ treatments for 6 h as outlined in Section 2.2. Subsequently, the insects were placed in an artificial climate chamber with ambient temperature for 30 min before being selected randomly and placed in a self-constructed insect-rearing jar and fed. The leaves of kidney bean plant with 50 individual 3rd-instar stage nymphs of *B. tabaci* were changed every 24 h. Survival days of *E. formosa* were recorded under different treatments, while the daily parasitism of *B. tabaci* nymphs was observed, and the number of parasitized nymphs was recorded. Each experiment was replicated three times.

Short-term extreme temperature treatment of the egg–larvae and pupae:

Additionally, 30 egg–larvae and 30 pupae of *E. formosa* were also exposed to extreme temperatures of HLT_25_, HLT_50_, LLT_25_, and LLT_50_ treatments for 6 h, as specified in Section 2.2. After treatment, the pupae were transferred to a self-constructed insect-rearing jar placed in an artificial climate chamber with ambient temperature for further development. The petiole was kept moist with water, and observations were made every 24 h. The eclosion date and the eclosion number of the emerged *E. formosa* were also recorded. Each experiment was repeated three times. A total of 30 adults within 24 h of emergence from each group were randomly selected and reared in the self-constructed insect-rearing jar in each group. The leaves of the kidney bean plant with 50 individual 3^rd^-instar stage nymphs of *B. tabaci* were replaced every 24 h. The survival days of *E. formosa* under the different treatments were recorded, the daily parasitism of the *B. tabaci* nymphs was observed, and the number of parasitized nymphs was recorded (Wang et al., 2018).

### 2.4 Effects of the short-term extreme temperature treatment on the growth and development of the F_1_ generation of *Encarsia formosa*

The leaves of *B. tabaci* nymphs were parasitized by *E. formosa* adults, and ten of these leaves were randomly selected for the respective temperature treatments outlined in Section 2.3. The emergence date and number of pupae that emerged were recorded every 24 h for the F_1_ generation of *E. formosa*. After pupation, each leaf was placed separately in a self-constructed insect-rearing jar, and the number of males and females and the emergence date of the F_1_ generation of *E. formosa* adults were recorded every 24 h. A total of 30 F_1_ generation *E. formosa* adults were randomly selected within 24 h of emergence and reared in the self-constructed insect-rearing jar. Leaves with 50 3^rd^-instar nymphs of *B. tabaci* were replaced every 24 h until the F_1_ generation *E. formosa* adults died, and the time and number of deaths were recorded (Wang et al., 2019).

### 2.5 Data analyses

The LT_25_ and LT_50_ values of *E. formosa* at various developmental stages, under short-term exposure to extreme temperature stress, along with associated experimental data on growth and development, were analyzed using one-way analysis of variance (one-way ANOVA) in SPSS 22.0. Tukey’s multiple-comparison test was utilized to identify significant differences in the relevant parameters of *E. formosa* at different developmental stages, following short-term exposure to different extreme temperatures. Survival rates were compared using the Kaplan-Meier procedure. Graphs representing the number of parasitism and survival rate were generated using Origin 2018.

## 3 Results

### 3.1 Corrected mortality of *Encarsia formosa* under short-term temperature stress

When subjected to short-term high-temperature stress for 6 h, *E. formosa* exhibited increasing corrected mortality at each developmental stage (egg-larvae, pupae, and adults) as the temperature increased, with no survival observed at 50°C. The corrected mortality of egg-larvae exceeded 40% when exposed to temperatures above 38°C, while adults could not survive at temperatures of 46 °C and above. In contrast, pupae demonstrated a strong ability to withstand high temperatures, surviving under short-term temperature stress ranging from 30°C to 46°C (egg-larvae: *F*_5,12_ = 241.67, *p* = 0.0001; pupae: *F*_5,12_ = 1180.90, *p* = 0.0001; adults: *F*_5,12_ = 289.93, *p* = 0.0001) (Table 1).

**TABLE 1:** Corrected mortality of the different developmental stages of *Encarsia formosa* (host: *Bemisia tabaci*) under short-term extreme temperature stress.

Temperature (°C)		Corrected mortality (%)		
		Egg–larvae	Pupa	Adult
High temperature	30	4.04 ± 0.18 D	0.00 ± 0.00 B	10.00 ± 1.93 E
	34	11.63 ± 1.17 D	0.00 ± 0.00 B	22.22 ± 2.94 D
	38	46.98 ± 4.91 C	0.00 ± 0.00 B	37.78 ± 2.22 C
	42	69.58 ± 4.10 B	5.56 ± 1.11 B	81.11 ± 4.01 B
	46	98.79 ± 0.95 A	93.33 ± 3.33 A	100.00 ± 0.00 A
	50	100.00 ± 0.00 A	100.00 ± 0.00 A	100.00 ± 0.00 A
Low temperature	−12	100.00 ± 0.00 a	85.56 ± 2.94 a	100.00 ± 0.00 a
	−8	98.26 ± 0.70 a	17.78 ± 2.22 b	82.22 ± 2.94 b
	−4	55.13 ± 2.88 b	4.44 ± 1.11 c	21.11 ± 1.11 c
	0	13.61 ± 1.04 c	0.00 ± 0.00 c	12.22 ± 2.22 d
	4	5.54 ± 0.85 d	0.00 ± 0.00 c	7.78 ± 1.11 d
	8	1.59 ± 0.41 d	0.00 ± 0.00 c	5.56 ± 1.11 d

Note: The data are presented as means ± SE., The different capital letters in the same column denote the significant difference within the same developmental stages in the high-temperature groups after treatment, based on the results of Tukey’s test (*p* < 0.05). The different lowercase letters in the same column denote the significant difference within the same developmental stages inthe low-temperature groups after treatment, based on the results of Tukey’s test (*p* < 0.05).

Conversely, when subjected to short-term low-temperature stress for 6 h, *E. formosa* exhibited increasing corrected mortality at each developmental stage as the temperature decreased. The corrected mortality of egg-larvae and adults exceeded 10% when exposed to temperatures at or below 0°C, with no survival recorded at temperatures below −12°C. Pupae exhibited higher tolerance to low temperatures than the other developmental stages, with survival recorded at temperatures ranging from −12°C to 8°C (egg-larvae: *F*_5,12_ = 1159.3, *p* = 0.0001; pupae: *F*_5,12_ = 463.28, *p* = 0.0001; adults: *F*_5,12_ = 605.09, *p* = 0.0001) (Table 1).

The HLT_25_ and HLT_50_ of the pupae were the highest at 42.87°C and 44.55°C, respectively, followed by the HLT_25_ and HLT_50_ of the egg–larvae at 35.77°C and 38.73°C, respectively. The HLT_25_ and HLT_50_ of the adults were the lowest at 33.96°C and 37.67°C, respectively. The LLT_25_ and LLT_50_ of the pupae were the lowest at −8.15°C and −9.78°C, respectively, followed by the LLT_25_ and LLT_50_ of the adults at −1.43°C and −4.78°C, respectively. The LLT_25_ and LLT_50_ of the egg–larvae were the highest at −0.45°C and −2.87°C, respectively (Table 2).

**TABLE 2:** HLT_25_, HLT_50_, LLT_25_, and LLT_50_ of the different developmental stages of *Encarsia formosa* (host: *Bemisia tabaci*) under short-term extreme temperature stress.

Handling stages	Regression equation	*R* ^2^	Different mortality temperature (°C)		95% Confidence interval
Egg–larvae	y = 0.01x^2^–0.53x+4.98	0.959	HLT_25_	35.77	34.19∼36.98
			HLT_50_	38.73	37.56∼39.94
	y = 0.02x^2^–0.26x-1.04	0.985	LLT_25_	−0.45	−1.52∼0.91
			LLT_50_	−2.87	−3.95∼-1.82
Pupa	y = 0.07x^2^–5.24x+99.42	0.974	HLT_25_	42.87	41.79∼43.61
			HLT_50_	44.55	43.83∼45.32
	y = 0.04x^2^+0.04x-0.63	0.982	LLT_25_	−8.15	−8.91∼-7.15
			LLT_50_	−9.76	−10.61∼-9.01
Adult	y = 0.01x^2^–0.52x+5.83	0.935	HLT_25_	33.96	32.57∼35.12
			HLT_50_	37.67	36.56∼38.85
	y = 0.02x^2^–0.12x-1.30	0.924	LLT_25_	−1.43	−3.25∼1.05
			LLT_50_	−4.78	−6.88∼-2.94

### 3.2 Lengths of the period of development of *Encarsia formosa* under short-term extreme temperature stress

Compared to other temperature treatments, the shortest duration for pupa-adult and egg–adult development (5.70 days and 12.65 days, respectively) was observed when egg–larvae of *E. formosa* were exposed to short-term treatment of HLT_25_. The duration of pupa-adult and egg-adult development increased when pupae were exposed to short-term treatment of HLT_25_ and HLT_50_, compared to the control (pupa–adult development at high temperature: *F*_4,45_ = 44.29, *p* = 0.0001; egg–adult development at high temperature: *F*_4,45_ = 33.07, *p* = 0.0001). Similarly, when egg-larvae and pupae were exposed to short-term treatments of LLT_25_ and LLT_50_, the duration of pupa–adult and egg-adult development was longer than that observed in the control (pupa-adult development at low temperature: *F*_4,45_ = 23.56, *p* = 0.0001; egg–adult development at low temperature: *F*_4,45_ = 16.39, *p* = 0.0001). The longest pupa–adult duration (8.24 days) was observed when egg-larvae were exposed to short-term treatment of LLT_50_, and the longest egg-adult duration (15.32 days) was observed when pupae were exposed to short-term treatment of LLT_50_ (Table 3).

**TABLE 3:** The period of development of each developmental stage of *Encarsia formosa* (host: *Bemisia tabaci*) under short-term extreme temperature stress.

Handling stages	Temperature (°C)	Period of development (d)			Eclosion rate (%)
		Egg-pupa	Pupa-adult	Egg- adult	
Control	25	7.10 ± 0.07 Aa	6.59 ± 0.12 Bb	13.69 ± 0.13 Bc	99.55 ± 0.46 Aa
Egg-larvae	HLT_25_ (35.77)	6.89 ± 0.08 A	5.70 ± 0.11 C	12.65 ± 0.12 C	97.07 ± 1.22 AB
	HLT_50_ (38.73)	6.93 ± 0.09 A	6.73 ± 0.08 B	13.67 ± 0.13 B	96.83 ± 1.38 AB
Pupa	HLT_25_ (42.87)	-	7.33 ± 0.08 A	14.43 ± 0.11 A	91.45 ± 2.59 B
	HLT_50_ (44.55)	-	7.37 ± 0.13 A	14.47 ± 0.15 A	71.53 ± 1.90 C
Egg-larvae	LLT_25_ (−0.45)	6.88 ± 0.15 a	7.75 ± 0.21 a	14.63 ± 0.23 b	96.95 ± 1.31 a
	LLT_50_ (−2.87)	6.85 ± 0.06 a	8.24 ± 0.11 a	15.08 ± 0.11 ab	95.38 ± 2.08 a
Pupa	LLT_25_ (−8.15)	-	8.03 ± 0.12 a	15.12 ± 0.12 a	82.30 ± 4.28 b
	LLT_50_ (−9.76)	-	8.22 ± 0.15 a	15.32 ± 0.18 ab	67.17 ± 3.58 c

Note: The data are presented as means ± SE., The different capital letters in the same column indicate significant differences in different developmental stages after high-temperature treatment, based on the results of Tukey’s test (*p* < 0.05). The different lowercase letters in the same column indicate significant differences in different developmental stages after low-temperature treatment, based on the results of Tukey’s test (*p* < 0.05).

The eclosion rates of egg–larvae and pupae were lower after short-term exposure to extreme temperatures than the control. The eclosion rates decreased with an increase in extreme-high temperature and a decrease in extreme-low temperature. The eclosion rates for egg–larvae exposed to short-term treatments of HLT_25_, HLT_50_, LLT_25_, and LLT_50_ were 97.07%, 96.83%, 96.95%, and 95.38%, respectively, which were not significantly different from the control. However, the eclosion rates of pupae exposed to HLT_25_ and HLT_50_ treatments were 91.45% and 71.53%, respectively, which were significantly lower than the control (*F*_4,45_ = 44.93, *p* = 0.0001). The eclosion rates of pupae exposed to LLT_25_ and LLT_50_ treatments were 82.30% and 67.17%, respectively, which were significantly lower than the control (*F*_4,45_ = 24.52, *p* = 0.0001) (Table 3).

### 3.3 Adult longevity and parasitism of *Encarsia formosa* under short-term extreme temperature stress

**FIGURE 1:**
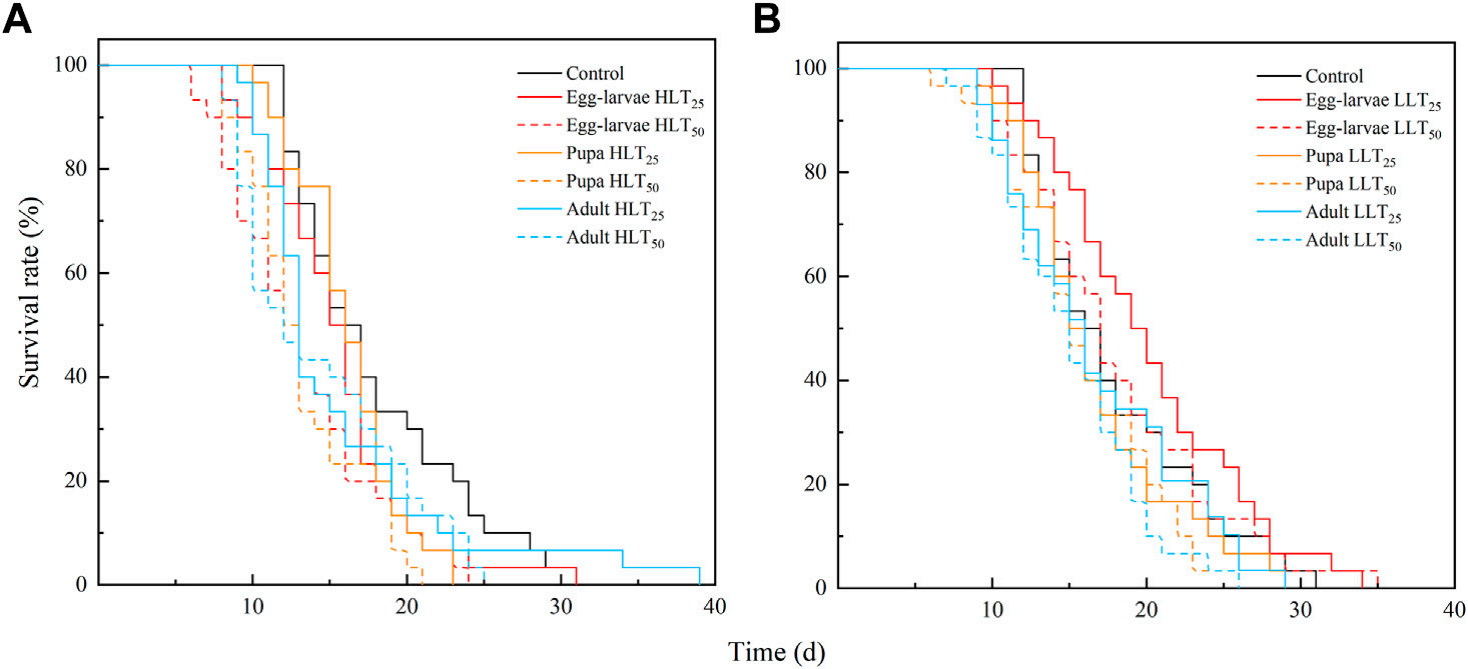
Survival rate of adult of *Encarsia formosa* (host: *Bemisia tabaci*) under short-term extreme temperature stress at each developmental stage. Note: Fig. **(A)** presents the survival rate of adult of *Encarsia formosa* under short-term high-temperature stress at each developmental stage. Fig. **(B)** presents the survival rate of adult of *E. formosa* under short-term low-temperature stress at each developmental stage.

The study employed Kaplan-Meier survival analysis to compare the survival rates of *E. formosa* at different developmental stages after short-term extreme temperature treatments. The results indicated that *E. formosa* adults experienced increased mortality between the 12th and 20th day (Figure 1). After exposure to HLT_50_, the adult longevity of *E. formosa* was significantly reduced to 13.07 days for egg-larvae and 13.27 days for pupae, compared to the control group (*F*_6,203_ = 3.29, *p* = 0.0042). However, the adult longevity was prolonged to 18.47 days for egg-larvae under the short-term treatment of LLT_25_ but shortened to 16.03 days under LLT_50_. The adult longevity was also reduced after exposure of pupae and adults to LLT_25_ and LLT_50_ treatments, with the shortest adult longevity (15.07 days) occurring after the adults were subjected to LLT_50_ (Table 4).

**TABLE 4:** Effects of short-term extreme temperature stress on the parasitic ability of *Encarsia formosa* (host: *Bemisia tabaci*).

Handling stages	Temperature (°C)	Longevity (d)	Number of parasitism (individual)
Control	25	17.83 ± 1.01 Aab	185.23 ± 15.01 Aa
Egg-larvae	HLT_25_ (35.77)	15.47 ± 0.88 AB	85.37 ± 7.18 C
	HLT_50_ (38.73)	13.07 ± 0.91 B	81.13 ± 5.51 C
Pupa	HLT_25_ (42.87)	16.13 ± 0.61 AB	89.77 ± 3.25 C
	HLT_50_ (44.55)	13.27 ± 0.71 B	41.20 ± 1.82 D
Adult	HLT_25_ (33.96)	15.53 ± 1.24 AB	160.60 ± 10.24 AB
	HLT_50_ (37.67)	14.27 ± 1.01 AB	136.73 ± 11.89 B
Egg-larvae	LLT_25_ (−0.45)	18.47 ± 1.12 a	125.87 ± 4.87 cd
	LLT_50_ (−2.87)	16.03 ± 1.12 ab	89.37 ± 7.54 d
Pupa	LLT_25_ (−8.15)	16.63 ± 0.92 ab	167.90 ± 10.66 ab
	LLT_50_ (−9.76)	15.73 ± 0.92 b	163.60 ± 8.47 ab
Adult	LLT_25_ (−1.43)	16.73 ± 1.05 ab	159.63 ± 5.88 ab
	LLT_50_ (−4.78)	15.07 ± 1.01 b	132.40 ± 7.54 bc

Note: The data are presented as means ± SE., The different capital letters in the same column indicate significant differences in different developmental stages after high-temperature treatment, based on the results of Tukey’s test (*p* < 0.05). The different lowercase letters in the same column indicate significant differences in different developmental stages after low-temperature treatment, based on the results of Tukey’s test (*p* < 0.05).

Furthermore, the total amount of parasitism decreased for all developmental stages of *E. formosa* under short-term extreme temperature stress. Egg–larvae were affected the most, followed by pupae and adults. Compared to the control group, the total number of adults with parasitism decreased significantly after egg–larvae were subjected to HLT_25_ and HLT_50_ treatments (85.37 individuals and 81.13 individuals, respectively), while pupae exposed to HLT_25_ and HLT_50_ treatments yielded 89.77 individuals and 41.20 individuals, respectively. Table 4 shows that the number of adults with parasitism was significantly lower in the HLT_50_ treatment group compared to the control group (136.73 individuals) (*F*_6,203_ = 31.88, *p* = 0.0001). Additionally, the number of adults with parasitism was lower in the LLT_25_ treatment group (125.87 individuals) and the LLT_50_ treatment group (89.37 individuals) compared to the control group. The number of adults with parasitism was also lower in the LLT_50_ treatment group (132.40 individuals) compared to the control group. These results indicate that the short-term exposure to extreme temperatures had a negative effect on parasitism. However, there was no significant difference in the total amount of parasitism after other treatments compared to the control group (*F*_6,203_ = 12.64, *p* = 0.0001) (Table 4).

**FIGURE 2:**
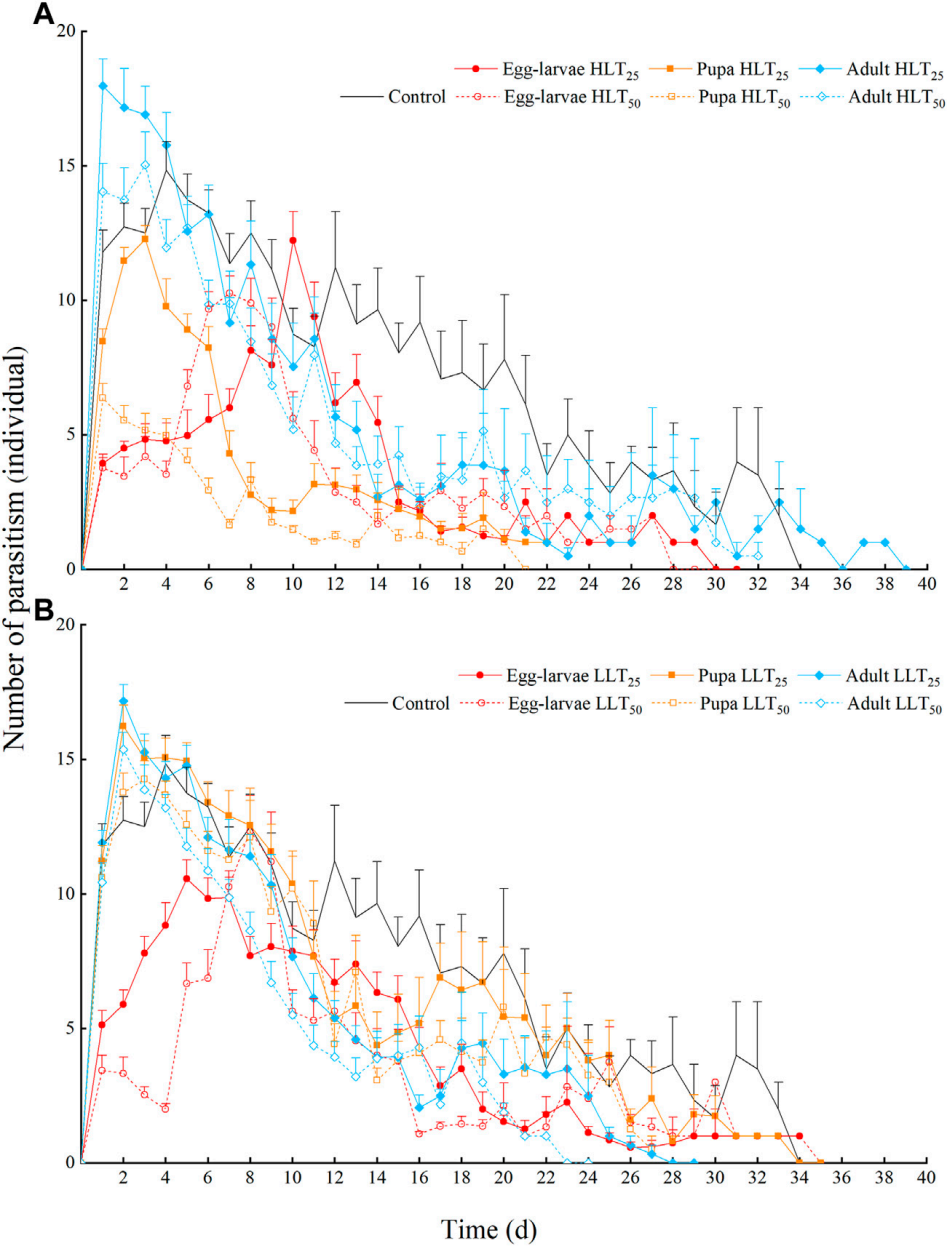
The daily number of adults with parasitism for *Encarsia formosa* (host: *Bemisia tabaci*) under short-term extreme temperature stress at each developmental stage. Note: The data are presented as means + SE. Fig. **(A)** presents the daily number of adults with parasitism for *Encarsia formosa* under short-term high-temperature stress at each developmental stage. Fig. **(B)** presents the daily number of adults with parasitism for *Encarsia formosa* under short-term low-temperature stress at each developmental stage.

The parasitism peaks were more concentrated and prominent in the short-term temperature stress groups of HLT_25_, HLT_50_, LLT_25_, and LLT_50_. The peaks were followed by a rapid decline in all groups. The parasitic peak of the control group was 14.83 individuals on the 4th day after emergence, followed by a relatively gentle downward trend. Exposure of pupae and adults to short-term extreme temperature stress resulted in earlier parasitism peaks. The parasitic peaks of pupae under HLT_25_, HLT_50_, LLT_25_, and LLT_50_ treatments, which were 12.27 individuals, 6.37 individuals, 16.23 individuals, and 14.27 individuals, respectively, occurred on the 3rd, 1st, 2nd, and 3rd day after emergence. Similarly, the parasitic peaks of adults after HLT_25_, HLT_50_, LLT_25_, and LLT_50_ treatments, which were 17.97 individuals, 15.03 individuals, 17.17 individuals, and 15.37 individuals, respectively, occurred on the 1st, 3rd, 2nd, and 2nd day after emergence. Finally, the parasitic peaks of egg–larvae after HLT_25_, HLT_50_, LLT_25_, and LLT_50_ treatments, which were 12.22 individuals, 10.27 individuals, 10.57 individuals, and 12.33 individuals, respectively, were delayed until the 10th, 7th, 5th, and 8th day after emergence, respectively (Figure 2).

### 3.4 Period of development and adult longevity of the F_1_ generation of *Encarsia formosa* under short-term extreme temperature stress

The development time of the F_1_ generation of *E. formosa* was prolonged after egg-larvae of *E. formosa* were subjected to short-term extreme high-temperature stress treatment. The development time of the F_1_ generation of *E. formosa* was shortened after the pupae were subjected to a short-term extreme high temperature stress treatment. In addition, the development time of the F_1_ generation of *E. formosa* at the same developmental stage under extremely high-temperature stress shortened with the increase of stress temperature. After egg–larvae were subjected to the short-term treatments with HLT_25_ and HLT_50_, the egg–pupa development time of the F_1_ generation was longer than that of the control, with values of 7.90 days and 7.80 days, respectively. The pupa–adult development time was the shortest (5.78 days) for the F_1_ generation after the pupa was subjected to the short-term treatment with HLT_25_. After the egg–larvae were subjected to the short-term treatment with LLT_25_ and the pupa was subjected to the short-term treatment with LLT_50_, the developmental duration of the F_1_ generation between egg and adult was 15.49 days and 11.98 days, respectively. These values were significantly different from the value of 13.69 days observed for the control (developmental time of egg–adult of the F_1_ generation: *F*_6,63_ = 13.53, *p* = 0.0001) (Table 5).

**TABLE 5:** The period of development of the F_1_ generation of *Encarsia formosa* (host: *Bemisia tabaci*) under short-term high-temperature stress at each developmental stage.

Handling stages	Temperature (°C)	Period of development (d)			Eclosion rate (%)	Female proportion (%)
		Egg-pupa	Pupa-adult	Egg- adult		
Control	25	7.10 ± 0.07 ab	6.59 ± 0.09 ab	13.69 ± 0.13 bc	99.55 ± 0.46 a	100.00 ± 0.00
Egg-larvae	HLT_25_ (35.77)	7.90 ± 0.23 a	7.59 ± 0.15 a	15.49 ± 0.15 a	98.33 ± 1.67 a	100.00 ± 0.00
	HLT_50_ (38.73)	7.80 ± 0.20 a	7.39 ± 0.16 a	15.19 ± 0.23 ab	97.98 ± 1.36 a	100.00 ± 0.00
Pupa	HLT_25_ (42.87)	6.70 ± 0.45 ab	5.78 ± 0.50 c	12.48 ± 0.52 cd	96.94 ± 1.57 a	100.00 ± 0.00
	HLT_50_ (44.55)	6.10 ± 0.42 b	5.88 ± 0.40 bc	11.98 ± 0.53 d	96.64 ± 2.06 a	56.38 ± 4.92
Adult	HLT_25_ (33.96)	6.70 ± 0.15 ab	6.98 ± 0.34 ab	13.68 ± 0.31 bc	98.69 ± 0.87 a	100.00 ± 0.00
	HLT_50_ (37.67)	6.70 ± 0.21 ab	6.72 ± 0.16 ab	13.42 ± 0.34 cd	97.92 ± 1.13 a	100.00 ± 0.00

Note: Data are means ± SE., Different letters in the same column denote significant difference among treatment different developmental stages (egg-larva, pupa, adult) within different groups after treatment by Tukey test (*p* < 0.05).

The developmental time of the F_1_ generation was prolonged after egg–larvae and adults of *E. formosa* were briefly exposed to extreme low-temperature stress. At the same developmental stage, the F_1_ developmental duration of *E. formosa* under extremely low temperature stress was shortened with the decrease in stress temperature. The egg–pupa development time of the F_1_ generation was shorter than that of the control after the pupa was exposed to the short-term treatments of LLT_25_ and LLT_50_, with the values of 6.90 days and 6.70 days observed for the former generation, respectively. The development time of pupa–adult and egg–adult of F_1_ generation was 7.74 days and 15.74 days, respectively, and these values were significantly higher than that of the control after the egg–larvae were subjected to the short-term treatment of LLT_25_ (development time of pupa-adult of F_1_ generation: *F*_6,63_ = 4.29, *p* = 0.0011; development time egg-adult of F_1_ generation: *F*_6,63_ = 8.95, *p* = 0.0001) (Table 6).

**TABLE 6:** The period of development of the F_1_ generation of *Encarsia formosa* (host: *Bemisia tabaci*) under short-term low-temperature stress at each developmental stage.

Handling stages	Temperature (°C)	Period of development (d)			Eclosion rate (%)
		Egg-pupa	Pupa-adult	Egg- adult	
Control	25	7.10 ± 0.07 ab	6.59 ± 0.09 b	13.69 ± 0.13 bc	99.55 ± 0.46 a
Egg-larvae	LLT_25_ (−0.45)	8.00 ± 0.21 a	7.74 ± 0.18 a	15.74 ± 0.18 a	99.23 ± 0.77 a
	LLT_50_ (−2.87)	7.60 ± 0.34 ab	6.98 ± 0.20 ab	14.58 ± 0.18 b	97.98 ± 1.36 a
Pupa	LLT_50_ (−8.15)	6.90 ± 0.18 b	7.24 ± 0.27 ab	14.14 ± 0.19 bc	95.60 ± 1.97 a
	LLT_50_ (−9.76)	6.70 ± 0.26 b	6.63 ± 0.14 b	13.33 ± 0.33 c	94.42 ± 2.15 a
Adult	LLT_50_ (−1.43)	7.20 ± 0.25 ab	6.87 ± 0.17 b	14.07 ± 0.35 bc	97.26 ± 1.40 a
	LLT_50_ (−4.78)	7.00 ± 0.26 ab	6.96 ± 0.22 ab	13.96 ± 0.36 bc	95.76 ± 2.26 a

Note: Data are means ± SE., Different letters in the same column denote significant difference among treatment different developmental stages (egg-larva, pupa, adult) within different groups after treatment by Tukey test (*p* < 0.05).

The eclosion rates of the F_1_ generation were lower at different developmental stages of *E. formosa* under short-term extreme temperature stress compared to the control. However, there was no significant difference among the treatments (short-term extreme high-temperature stress: *F*_6,63_ = 0.57, *p* = 0.7565; short-term extreme low-temperature stress: *F*_6,63_ = 1.44, *p* = 0.2125). The emergence rate of the F_1_ generation after pupae were subjected to short-term extreme temperature stress was the lowest and did not exceed 97%. In the F_1_ generation of *E. formosa*, male adults appeared after pupae were exposed to the short-term treatment of HLT_50_, while the proportion of females was just 56.38%. In contrast, male individuals did not appear in the other short-term temperature treatments (Table 5; 6).

**FIGURE 3:**
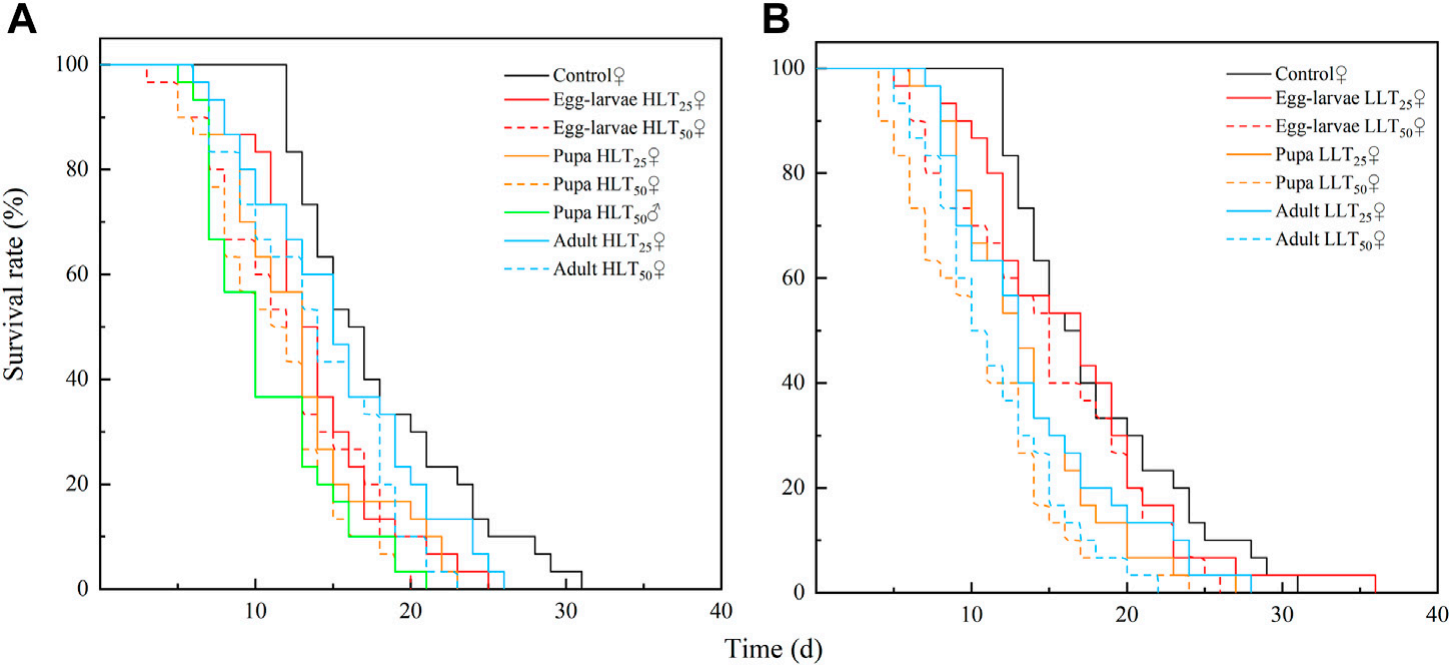
Survival rate of the F_1_ adult of *Encarsia formosa* (host: *Bemisia tabaci*) under short-term extreme temperature stress at each developmental stage. Note: Fig. **(A)** presents the adult survival rate of the F_1_ adult of *Encarsia formosa* under short-term high-temperature stress at each developmental stage. Fig. **(B)** presents the adult survival rate of the F_1_ adult of *E. formosa* under short-term low-temperature stress at each developmental stage.

**FIGURE 4:**
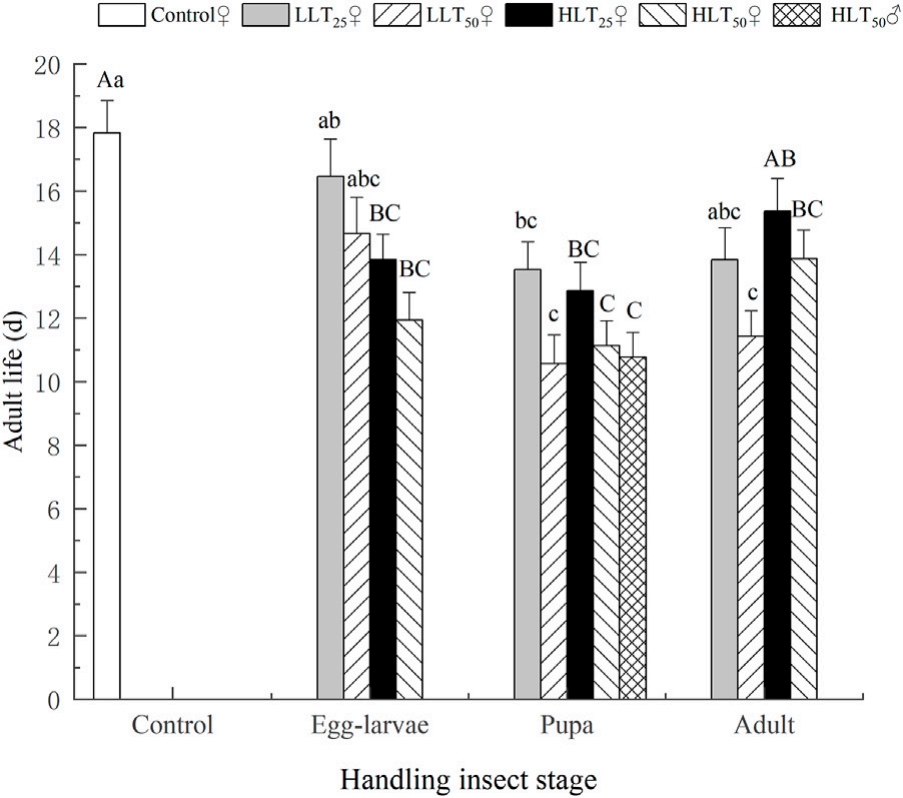
Adult longevity of the F_1_ generation of *Encarsia formosa* (host: *Bemisia tabaci*) under short-term extreme temperature stress at each developmental stage. Note: The different capital letters above the bars denote significant differences within the same developmental stages in the high-temperature groups after treatment, based on the results of Tukey’s test (*p* < 0.05). The different lowercase letters above the bars denote significant differences within the same developmental stages in the low-temperature groups after treatment, based on the results of Tukey’s test (*p* < 0.05).

After short-term extreme temperature stress at different developmental stages of *E. formosa*, the death of F_1_ generation adults of *E. formosa* occurred mainly on the 12th–20th day, and the survival rate showed a significant downward trend (Figure 3). The adult longevity of the F_1_ generation was 13.07 days and 13.27 days after egg–larvae were subjected to the short-term treatments of HLT_25_ and HLT_50_. The female adult longevity of the F_1_ generation was 12.87 days and 11.13 days, respectively, after the pupae were subjected to the short-term treatments of HLT_25_ and HLT_50_. The male adult longevity of the F_1_ generation was 10.77 days after the pupae were subjected to the short-term treatment of HLT_50_. Additionally, the adult longevity of the F_1_ generation was 13.87 days after the adults were subjected to the short-term treatment of HLT_50_, and this value was significantly shorter than the corresponding value for the control (*F*_7,232_ = 6.92, *p* = 0.0001). The adult longevity of the F_1_ generation was not significantly different from that of the control after exposure of egg–larvae to the LLT_25_ and LLT_50_ treatments and also after exposure of adults to the LLT_25_ treatment. Conversely, the adult longevity of the F_1_ generation was significantly lower than that of the control after exposure of pupae to the LLT_25_ and LLT_50_ treatments, with values of 13.53 days and 10.57 days, respectively. Additionally, the adult longevity of the F_1_ generation was 11.43 days after the adults were subjected to the short-term treatment of LLT_50_, and this value was also significantly lower than that of the control (*F*_6,203_ = 6.68, *p* = 0.0001) (Figure 4).

## 4 Discussion

Insects have a specific temperature range suitable for their growth, and temperatures beyond this range can inhibit their normal life activities and even result in death (Bowler and Terblanche, 2008). The tolerance of insects to temperature varies at different developmental stages or instar stages (Kingsolver et al., 2011). The Bogert effect hypothesis suggests that non-mobile stages (eggs and pupae) have greater heat resistance than mobile stages (adults and larvae), as the latter can escape the harmful environment and prevent damage from high temperatures (Huey et al., 2003). Studies have shown that insect pupae contain cold-resistant substances, such as glycerol, sorbitol, and trehalose, enabling greater cold tolerance than other stages (Feng et al., 2018). The present study found that short-term exposure to extreme temperatures significantly affected the corrected mortality of *E. formosa* at different developmental stages. The sequence of HLT_25_ and HLT_50_ under short-term high-temperature stress was pupa > egg–larva > adult, while the sequence of LLT_25_ and LLT_50_ under short-term low-temperature stress was pupa < adult < egg–larva. These results indicate that the egg–larvae and adults of *E. formosa* are less tolerant to short-term extreme temperature stress. Previous studies have also reported that different developmental stages of insects exhibit different tolerances to extreme temperatures. For example, Zhang et al. (2015) found that the pupa of *Plutella xylostella* (Linnaeus) (Lepidoptera: Plutellidae) had a higher LT_50_ value than the egg, young larvae, and adults. Yang et al. (2021) reported that the pupa of *B. tabaci* had greater resistance to high temperatures compared to the egg, larva, and adult. Furthermore, pupae of *Spodoptera frugiperda* (Smith) (Lepidoptera: Noctuidae) were found to be the most tolerant to low temperatures, with a higher survival rate than other stages at −10°C and −5°C for 2 h (Zhang et al., 2021). Similarly, in this study, pupae of *E. formosa* were found to have the strongest tolerance to extreme temperature stress, possibly due to their low mobility and ability to resist adverse effects through innate tolerance. Conversely, the egg–larva stage exhibited the weakest tolerance to low-temperature stress, likely due to the frostbite of plant leaves that resulted in reduced survival of *B. tabaci*, which, in turn, could not provide adequate nutrients to the larvae of *E. formosa*.

Temperature plays a critical role in insect growth, development, and reproduction (Sánchez-Guillén et al., 2016). As ambient temperature rises, insects can maintain their reproductive ability of the population under high-temperature stress by shortening their developmental duration up to a certain threshold. However, beyond this threshold, the period of insect development increases (Du et al., 2007). Conversely, low temperatures slow down the metabolic rate and activity of physiological substances in insects, inhibiting their growth and development, ultimately affecting the population’s development (Onalethata et al., 2021). In this study, short-term extreme high-temperature stress caused the egg–pupa and egg–adult development durations of *E. formosa* to shorten, while short-term extreme low-temperature stress prolonged the pupa–adult and egg–adult developmental durations. A higher stress temperature resulted in longer developmental duration. Previous studies have shown that the developmental period of various insect species is affected by temperature stress. For instance, the developmental duration of eggs, larvae, and pupae of *Leiometopon simyrides* Staudinger (Lepidoptera: Noctuidae) decreased with increasing temperature within the range of 18°C–30°C, but exposure to 33°C slightly increased the developmental duration of eggs, 3rd-instar, and 5th-instar larvae (Gou et al., 2022). In *S. frugiperda*, Zhang H M et al. (2020) found that exposing eggs and pupae to short-term high-temperature stress affected the growth and development of larvae and pupae, respectively. Huang et al. (2020) reported that the period of development of all stages of *Zeugodacus tau* (Walker) (Diptera: Tephritidae) and *Zeugodacus cucuribitae* (Coquillett) (Diptera: Tephritidae) increased as the applied low-temperature decreased. Furthermore, Yu et al. (2022) found that at low temperatures, both the egg and larval stages of *Bactrocera dorsalis* (Hendel) (Diptera: Tephritidae) developed significantly longer than the control group, with the egg stage prolonging by more than twice at 0°C. Similarly, the egg and larval stages of *Galeruca daurica* Joannis (Coleoptera: Chrysomelidae) developed for longer periods at low temperatures (Li et al., 2015). These results are consistent with the findings of this study, which suggest that extreme temperatures significantly affect the developmental period of *E. formosa* at each stage.

When ambient temperature exceeds the optimal range for insect development, energy is devoted towards enhancing tolerance to extreme temperatures, thereby improving survival rate and reducing adult longevity and reproduction (Jervis et al., 2005; Zhao et al., 2017). Wolbachia in parasitic wasps regulates host reproduction by inducing cytoplasmic incompatibility, parthenogenesis, male feminization, and male killing. Inactivation of Wolbachia occurs due to high-temperature stress or antibiotic treatment, which leads to male offspring (Stouthamer and Mak, 2020; Power et al., 2022). The present study showed that short-term exposure to extreme temperature significantly decreased adult longevity and total parasitism after the pupae and adults of *E. formosa* were subjected to high- or low-temperature stress. Male individuals appeared in the F_1_ generation after the pupae were subjected to the short-term treatment of HLT_50_. Aroosa et al. (2022) found that the survival rate and fecundity of green peach aphid [*Myzus persicae* (Sulzer) (Hemiptera: Aphididae)] were significantly lower than the control after treatment at 36°C for 10 h. Sarmad et al. (2020) reported that the mating probability, fecundity, and adult longevity of *Dysdercus koenigii* Fabricius (Heteroptera: Pyrrhocoridae) decreased with decreasing stress temperature after exposure to short-term extreme low-temperature stress. Wolbachia density in *Nasonia vitripennis* (Walker) (Hymenoptera: Pteromalidae) decreased significantly after treatment with critical temperatures, which affected the insect’s reproductive mode and offspring sex ratio (Yang et al., 2020). Zhou et al. (2009) reported that high-temperature shock affects Wolbachia-induced *E. formosa* reproduction by increasing the proportion of male offspring. Overall, short-term extreme temperature stress could affect parasitism ability and offspring sex ratio in *E. formosa*, thereby reducing the control effect of *E. formosa* against pests.

To avoid damage to *E. formosa* caused by extreme temperatures, weather changes should be observed when releasing *E. formosa* for field control, and extreme temperature weather should be avoided. During summer, greenhouse facilities should have proper ventilation to reduce the influence of high temperatures on *E. formosa* growth and offspring sex ratio. During extreme temperature weather, the release amount of *E. formosa* should be increased to achieve better control over *B. tabaci*.

## Data Availability

The original contributions presented in the study are included in the article/supplementary material, further inquiries can be directed to the corresponding author.
